# Lanthanide Centered and Circular Polarized Luminescence (CPL) From Tb(III) Complexes Made From Structurally Simple Methoxy‐Phenyl and Phenol‐Derived Pyridyldiamide (pda) Ligands

**DOI:** 10.1002/chem.71535

**Published:** 2026-08-11

**Authors:** Jennifer E. Jones, Jonathan A. Kitchen, Robert D. Peacock, Thorfinnur Gunnlaugsson

**Affiliations:** ^1^ School of Chemistry and Trinity Biomedical Sciences Institute Trinity College Dublin The University of Dublin Dublin Ireland; ^2^ Chemistry School of Science Faculty of Health and Environmental Sciences Auckland University of Technology Auckland New Zealand; ^3^ School of Chemistry University of Glasgow Glasgow Scotland UK

**Keywords:** chirality, CPL, lanthanide, luminescence, self‐assembly, Tb(III)

## Abstract

The chiral pda derived anisole ligands **L1a** [**1(*R,R***)] and **L1b** [**1(*S,S***)], along with their phenol derivatives **L2a** [**2(*R,R***)] and **L2b** [**2(*S,S***)], were used for lanthanide‐directed synthesis of their respective Tb(III) complexes. Under thermodynamic control, the 3:1 (ligand:metal) enantiomerically pure assemblies were formed, including the Tb(III) complex of **L1b** (*S,S*) which was structurally characterized by x‐ray crystallography. These Tb‐complexes exhibited Tb(III)‐centered emission upon ligand excitation, demonstrating efficient sensitization of their ^5^D_4_ excited states. Their self‐assembly formation under kinetic control was also investigated through monitoring the ground, the singlet and the Tb(III) excited state changes in situ and analyzed using non‐linear regression fitting of the changes in both the ground and the exited states. Moreover, their chiroptical properties were also probed by using both circular dichroism (CD) and circular polarized luminescence (CPL) spectroscopy, where all the complexes displayed signals of equal and opposite handiness for given enantiomers.

## Graphical abstract

The development of chiral pda derived methoxy ligands **L1a** [**1(*R,R***)] and **L1b** [**1(*S,S***)], along with their phenol derivatives **L2a** [**2(*R,R***)] and **L2b** [**2(*S,S***)], and their respective Tb(III) complexes is described. These Tb‐complexes exhibited Tb(III)‐centered emission upon and their chiroptical properties were probed by using both CD and CPL spectroscopy.

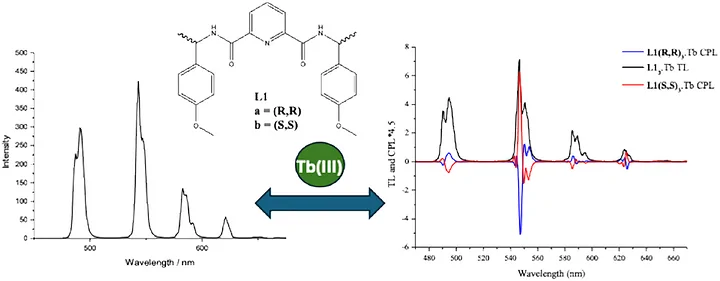

## 1 Introduction

Development of chiral lanthanide self‐assembly supramolecular structures has become an active area of research in recent times [1, 2, 3, 4, 5, 6, 7]. The lanthanides possess high coordination requirements, which allows for their use in directing the formation of self‐assembly structures such as bundles [8, 9, 10], helicates [11, 12, 13, 14, 15, 16, 17, 18, 19, 20], mechanically interlocked molecules [21, 22, 23], and knots [24, 25] and other higher order architectures [26, 27, 28, 29, 30, 31]. Many of the lanthanide ions have also been employed in the formation of higher order or functional soft‐materials [32, 33, 34, 35], cages [36, 37], and in coordination polymers and metal‐organic frameworks (MOFs) [38, 39]. We have developed several examples of ligands with sensitizing antennae and probed the self‐assembly formation between these and lanthanides by monitoring the photophysical changes of the resulting lanthanide directed self‐assembly formation in solution [19, 40, 41, 42, 43, 44, 45]. In particular, we have employed chiral tridentate ligands based on both 1‐ or 2‐naphthalene based 2,6‐pyridyldiamide (**pda**) [46, 47, 48], and 2,6‐bis(1,2,3‐triazol‐4‐yl)pyridine (**btp**) [49, 50] based ligands, as well chiral macrocyclic cyclen based systems [51]. We have also used this design strategy in the generation of responsive soft materials with self‐healing properties [52, 53]. In all of these chiral examples, and that of others, the proximity of the chiral center close to the binding site of the lanthanide ion manifests itself in the transfer of the chirality from the ligand to the lanthanide ion, resulting in the emission of circularly polarized lanthanide luminescence (CPL) [54, 55]. Leigh and co‐workers also have elegantly demonstrated the use of such ligands and lanthanides in the formation of molecular knots, using 1‐naphthalene analogues. This they did by synthetically installing functional group onto a naphthalene moiety that allowed for incorporation of the necessary chains to facility the ring closure using Grubb's catalysts [56]. Surprisingly, rarely are such synthetic modifications undertaken that allow for further derivatization at the ‘antennae’ site.

Herein, we describe our investigation into the use of chiral methoxy‐phenyl (these chiral anisoles are commercially available) derivatives of **pda,** the enantiomers **L1a** [**1(*R,R***)] and **L1b** [**1(*S,S***)], for use in the lanthanide directed synthesis, Scheme 1. The phenol derivatives of these ligands **L2a** [**2(*R,R***)] and **L2b** [**2(*S,S***)], Scheme 1, were then also formed by simple BBr_3_ mediated cleavage of the methoxy groups of the enantiomers **L1a** and **L1b**. Unlike our previous work (where we have demonstrated functionalization at the fourth position of **pda** unit) the ether moiety in these two ligands, can be viewed as a stepping stone toward the formation of more structurally complex systems, through direct functionalization at the hydroxy site. We demonstrate herein, using both solution and solid‐state chemistry, that the Tb(III) complexes of these ligands give rise to the formation of Ln(III) structures consisting of 3:1 (ligand:metal) stoichiometry. We have not employed Tb(III) previously in the development of chiral **pda**‐Ln complexes/assemblies. Tb(III) was selected for this work given that phenyl and anisole groups are known to be a good sensitizer for Tb(III) [51, 52, 53], as the Tb(III) ^5^D_4_ lies at 22,450 cm^−1^, while such phenyl based ligands have T_1_ state energy levels calculated by Zhan et al. [55] to be around 24,400 cm^−1^, which reduces back energy transfer from the ^5^D_4_ state. We also show that under kinetic control these ligands interact with Tb(III) through a stepwise **L‐Tb** formation, where in the excess of the Tb(III) the 1:1 stoichiometry dominates. Furthermore, the chirality of these lanthanide structures was probed using CPL spectroscopy [3, 4, 5, 6, 7, 54, 55], where the handedness of the lanthanide emission was monitored, upon excitation of the chiral antennae.

**SCHEME 1:**
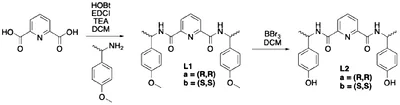
Synthesis of the two chiral (*R,R* or *S,S*) dpa ligands **L1a** and **L1b**, and the subsequent cleavage of the methoxy groups to give **L2a** and **L2b**.

## 2 Results and Discussion

### 2.1 Synthesis and Characterization of **L1a**, **L1b**, **L2a**, and **L2b**

The synthesis of **L1a** and **L1b** was achieved in a single step from commercially available starting materials, Scheme 1. Dipicolinic acid was reacted with the chiral (*R* or *S*) amine using peptide‐coupling, in the presence of triethylamine, HOBt and EDCI in dry DCM. This gave **L1a** [**1(*R,R***)] and **L1b** [**1(*S,S***)], Scheme 1, respectively. The reaction was monitored by TLC and after 48 h worked up. The organic solution was washed with NaHCO_3_(sat. aq.) solution, water and finally brine, dried over MgSO_4_, filtered and then evaporated to dryness. Trituration of the crude product in acetonitrile gave **L1a** and **L1b** in 54 and 48% yields, respectively. The successful ligand formation was confirmed using ^1^H NMR, ^13^C{^1^H} NMR, UV‐vis, CD and IR spectroscopy, as well as using HRMS and elemental analysis (See Supporting Information for details).

The ^1^H NMR of the two enantiomers is shown in Figure 1a (CDCl_3_), demonstrating that the two were formed as single products (See Supporting Information for expanded spectra and the ^13^C NMRs). The proton of the stereogenic center was observed at 5.3 ppm as a quartet, while the adjacent methyl group appeared as a doublet at 1.6 ppm. The methoxy group provided a singlet. The NMR spectra of **L1b** are identical to the ones of **L1a**, as shown in the Supporting Information. The ground state of the ligands was examined using UV–vis spectroscopy in spectroscopic grade acetonitrile at room temperature, giving rise to a broad absorption band centered at 275 nm, assigned to the anisole antenna group, as well as observing the π–π* transitions at *ca*. 224 nm of the pyridine chelating unit (Figure 2).

**FIGURE 1:**
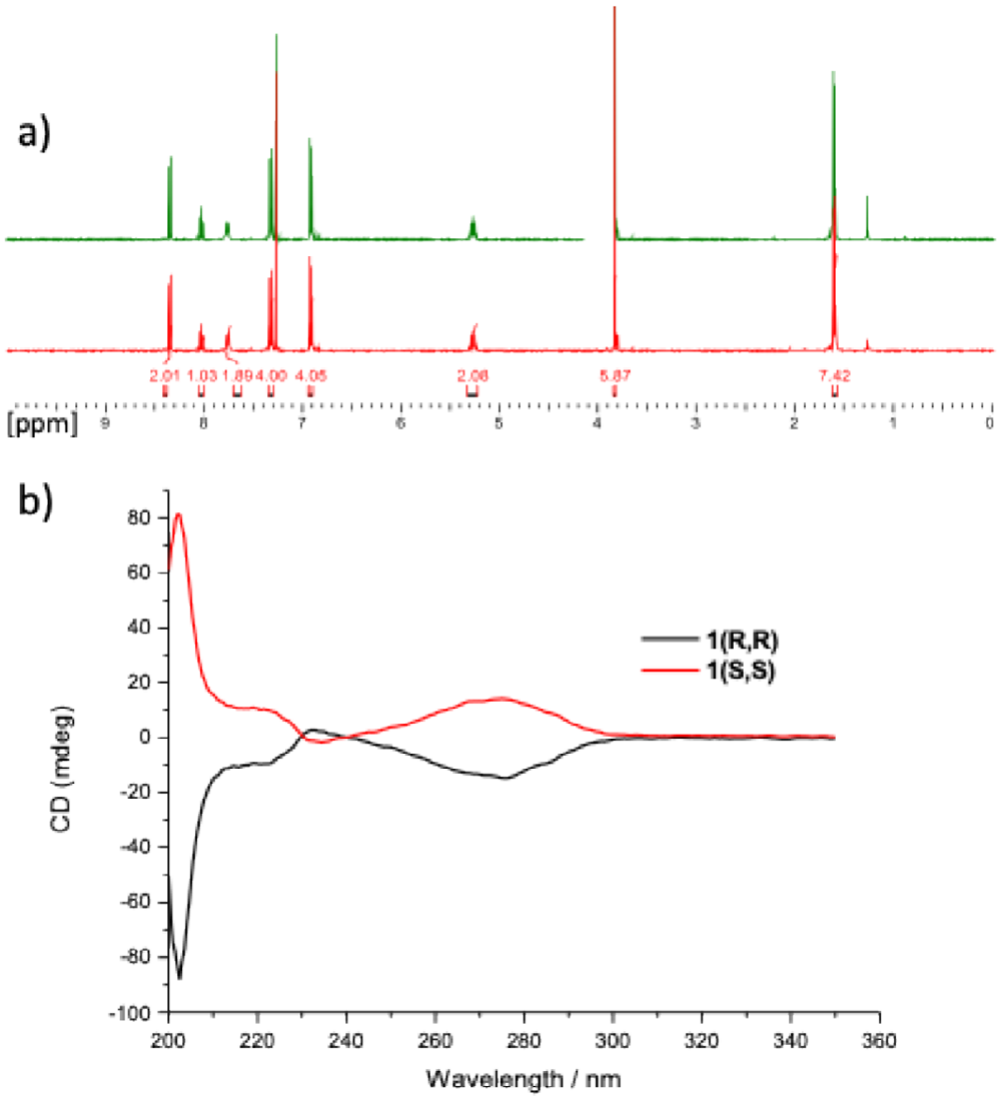
(a) The ^1^H NMR (CDCl_3_) of the **L1a** (*R,R* red) and **L1b** (*S,S*, green), respectively, (See Supporting Information for expanded versions). (b) The CD‐spectra of **L1a** (black) and **L1b** (red), respectively, when recorded in spectroscopic grade acetonitrile solution (2 × 10^−3^ mol dm^−3^) at room temperature.

**FIGURE 2:**
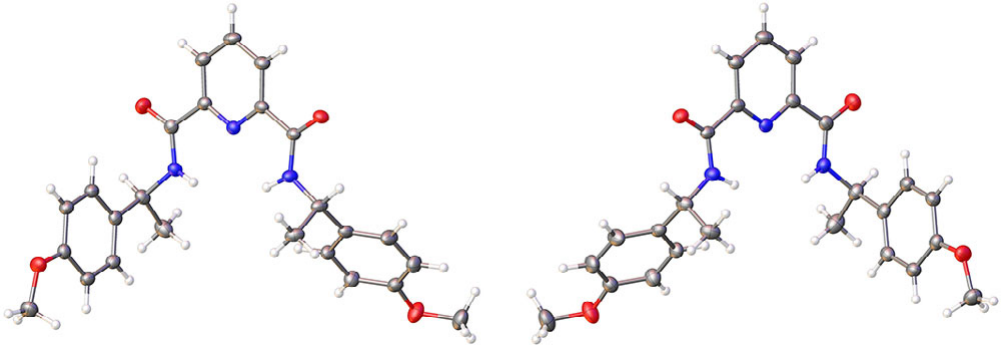
The solid‐state crystal structures of the two enantiomers of **L1a** (left); and **L1b** (right). Atoms shown as 50% probability ellipsoids: O (red), N (blue), and C (grey).

The use of CD‐spectroscopy confirmed that **L1a** and **L1b** were obtained as pair of enantiomers as is demonstrated in Figure 1b, where mirror‐image dichroism bands, of equal magnitude and opposite signs, were observed, consistent with the presence of a single chiral stereoisomer in solution. Here, the *S,S* isomer **L1a** gave rise to a positive Cotton effect for both the high energy, as well as the long wavelength transition, while the *R,R* isomer **L1a** gave the mirror result, with only minor crossover at *ca*. 233 nm. This is somewhat different from our work where generally a clear change of sign was observed in the dichroism bands for the higher vs. the lower energy transitions.

Colorless crystals of **L1a** and **L1b** were grown by slow evaporation of methanol and the low temperature (108 K) molecular structures determined, Figure 2. **L1a** crystallized in the chiral tetragonal space group *P*4_1_ while **L1b** crystallized in the enantiomorphic space group, *P*4_3_. Both contained one molecule in the asymmetric unit where **L1a** was the R enantiomer and **L1b** the *S* enantiomer. The anisole arms are orientated such that they adopt a trans type of configuration (i.e. when viewed from above the arms are oriented away from opposing faces of the pyridyl ring). The alternative would be a *cis*‐like conformation (*i.e*. when viewed from above the arms are oriented on one side of the pyridyl ring). The NH protons in **L1a** and **L1b** are orientated inwards toward the pyridyl nitrogen atom as seen with previous structures and are involved in hydrogen bonding interactions to a neighboring amide carbonyl oxygen atom [N(1)···O(3) = 2.920(2) Å, <(N(1)‐H(1)···O(3)) = 158(2)° and N(3)···O(3) = 2.981(2) Å, <(N(3)‐H(3)···O(3)) = 159(2)°] for **L1a** and [N(1)···O(2) = 2.978(2) Å, <(N(1)‐H(1)···O(2)) = 155(2)° and N(3)···O(2) = 2.919(2) Å, <(N(3)‐H(3)···O(2)) = 159(2)°] for **L1b**. The end packing result of these strong H‐bonding interactions is the formation of H‐bonded chains of molecules in the direction of the crystallographic c‐axis.

The two ligands **L1a** and **L1b** were also subjected to a deprotection of the methoxy groups, which was achieved by dissolving the desired compound in CH_2_Cl_2_, cooling to 0°C and adding an excess of BBr_3_. This solution was maintained at 0°C for 10 min and the reaction allowed to reach room temperature. After an hour, further BBr_3_ was added and the reaction left stirring at room temperature for 48 h. After this time, the reaction was treated to an aqueous basic workup. Confirmation of the successful formation of these ligands was achieved using ^1^H and ^13^C NMR (See SI; ^1^H NMR was recorded both in DMSO‐*d_6_* and CD_3_OD for comparison as these ligands were poorly soluble in CDCl_3_), IR, HRMS and microanalysis. The new phenolic protons were observed in the ^1^H NMR in the DMSO‐d_6_ spectra (See Supporting Information) as well as the coalescence of the NH protons with the pyridyl (*para*) proton to form a more complex multiplet signal (See Supporting Information). As above, both ligands **L2a** and **L2b** gave rise to CD spectra that were equal mirror images, closely resembling that seen for **L1** (See Supporting Information), indicating that this transformation did not affect the CD spectra much.

### 2.2 Synthesis and Characterization of Tb(III) Complexes of **L1** and **L2**

Having successfully made and structurally characterized the enantiomers of **L1**, we next set out to form complexes between these ligands and lanthanide ions. The focus here is on the use of Tb(III), but most of the work to date on such **bta** based chiral ligands has focused on the use of Eu(III). These ligands were designed containing tridentate binding pockets. However, as lanthanides have high coordination requirements, and based on our previous work using naphthalene antennae, the coordination requirement can only be perfectly matched using three such ligands. Hence, we generated Tb(III) complexes from **L1a** and **L1b** in 3:1 ligand to metal stoichiometry, as well as **L2a** and **L2b**.

The **L_3_:Tb** complexes of **L1**, were obtained by dissolving the ligand in a 3:1 ratio with Tb(III) triflate in methanol and heating the resulting mixture at 70°C for 20 min under microwave irradiation. The resulting clear solution was subjected to slow vapor diffusion with diethyl ether to yield colorless crystalline solids in 29–58% yields. The complexes were characterized by IR, ^1^H NMR and microanalysis. The ^1^H NMR of the products generated using **L1** were identical, illustrated the presence of six paramagnetic resonances between +38 ppm and −15 ppm, demonstrating the presence of a single (symmetric) paramagnetic species in solution, Figure 3 for **L1b** (See Supporting Information for both complexes). And that the two complexes were formed as enantiomers (with identical NMRs). Unfortunately, mass spectrometry was unsuccessful in characterizing the complex formation. However, the solid‐state structure was confirmed using x‐ray crystallography. The Tb(III) complex of ligand **L1b** was determined, however a Tb(III) complex of **L1a** could not be crystallized [54, 55] (*c.f*. caption Figure 4 and Supporting Information). Colorless crystals of [Tb(**L1b**)_3_](CF_3_SO_3_)_3_·H_2_O were grown by slow evaporation of methanol and the low temperature (162 K) structure determined. Crystals were emissive under UV irradiation showing characteristic green Tb(III) emission (See Supporting Infomation). [Tb(**L1b**)_3_](CF_3_SO_3_)_3_·H_2_O crystallized in the triclinic space group *P*1 and contained one complex molecule in the unit cell with one interstitial water molecule.

**FIGURE 3:**
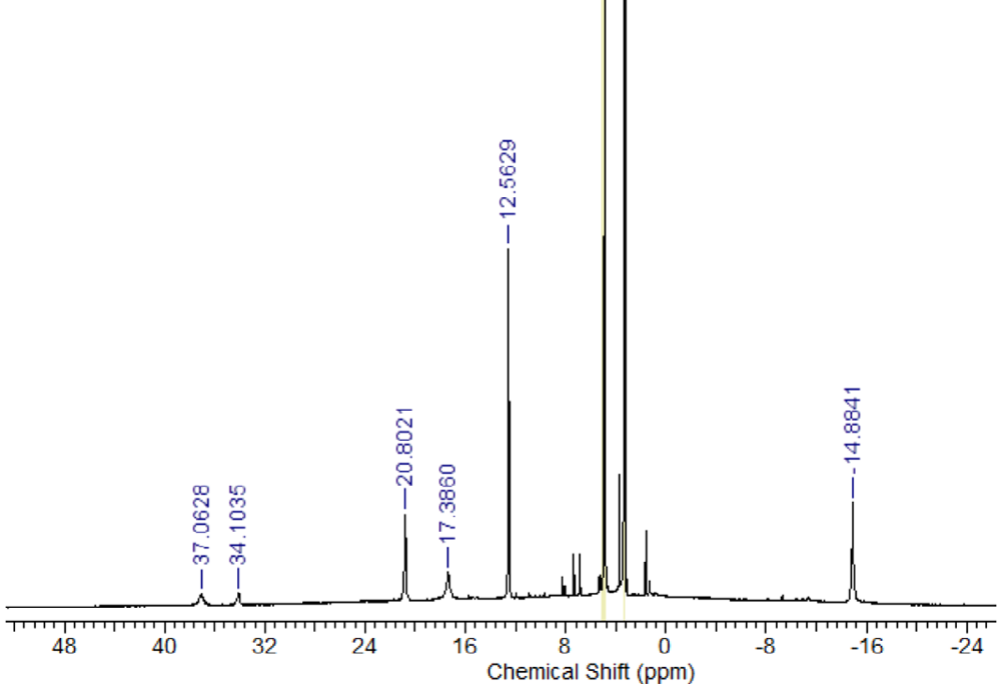
The ^1^H NMR (400 MHz) of 3:1 **1Lb**:Tb(III) in MeOD‐*d*_4_. See Supporting Information for both complexes.

**FIGURE 4:**
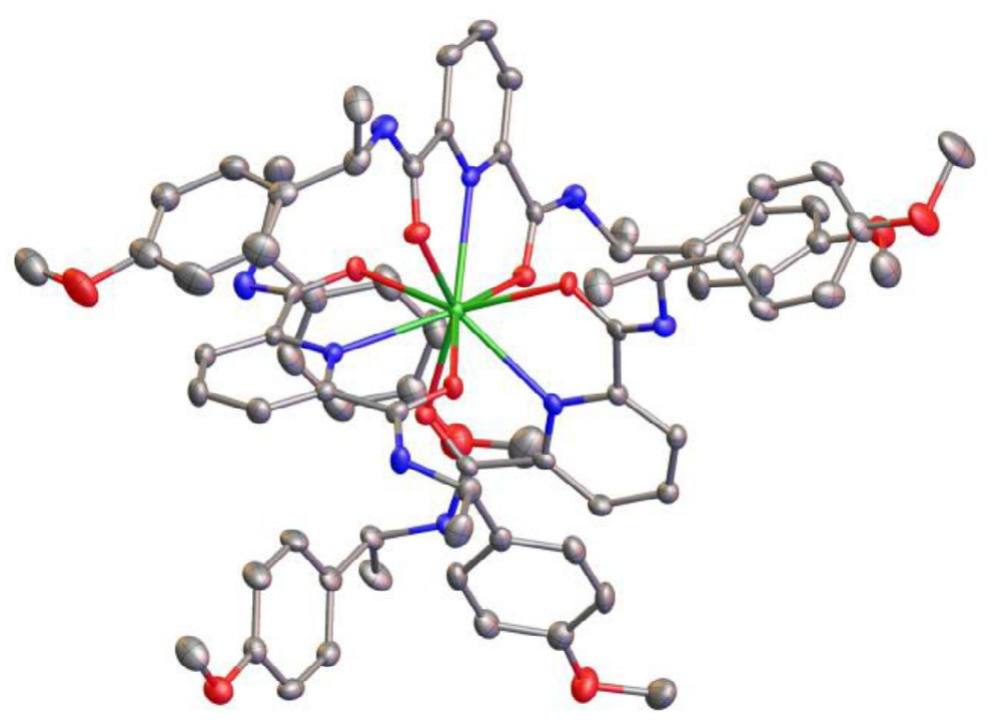
The molecular structure of the Tb(III) complex [Tb(**L1b**)_3_](CF_3_SO_3_)_3_·H_2_O formed using **L1** as determined by SCXRD. Anions, hydrogen atoms and interstitial solvent molecules have been omitted for clarity. Atoms shown as 50% probability ellipsoids: Tb (green), O (red), N (blue), and C (grey), giving rise to a Δ‐configuration. The Eu(III) complex of **L1a** enantiomer [Eu(**L1a**)_3_](ClO4)_3_·2PrOH, was also formed, as the Tb(III) of **L1a** did not yield crystals suitable for SCXRD quality. The structure is displayed in the SI for comparison and shows the Λ‐configuration.

The structure demonstrates that the three ligands are acting in a tridentate fashion and all coordinate through the pyridyl nitrogen atom and two amide oxygen atoms to give an overall N_3_O_6_ coordination sphere, again with a slightly distorted tricapped trigonal prismatic geometry. Bond lengths are consistent with structures of this type [Tb‐O range = 2.379(3) – 2.416(3) Å; Tb‐N range = 2.521(3) – 2.533(3) Å]. Again, it was observed that the chirality from ligand **L1b** is transferred to the metal center which adopts a Δ‐configuration. In [Tb(**L1b**)_3_] all three ligands adopt slightly different conformations with respect to the pendant arms, one has the trans‐configuration, one adopts the “horseshoe” cis‐configuration, and the final ligand is somewhat intermediate between the two (See Supporting Information). Again, packing interactions are heavily dominated by hydrogen bonding (See Table in Supporting Information) between the amide protons and the triflate counter anions and interstitial water molecule creating a complex H‐bonding network in the extended structure.

Tb(III) complexes of **L2** were formed in a similar manner to that described for **L1** complexes. Characterization data was consistent with formation of the 3:1 complex. The ^1^H‐NMR of [Tb(**L2a**)_3_](OTf)_3_ showed the expected range of resonances and the elemental analysis showed the isolation of the desired product.

### 2.3 Spectroscopic Studies of Tb(III) Complexes of **L1** and **L2**

Having synthesized and fully characterized the complexes, we next investigated their photophysical properties in acetonitrile. The absorption spectrum of [Tb**(L1b)_3_**.](OTf)_3_ gave rise to a high energy transition, and a longer wavelength band centered at 275 nm (See Supporting Information). Excitation at 275 nm yielded characteristic Tb(III) emission (the phosphorescence emission spectrum), demonstrating the successful population of the Tb(III) ^5^D_4_ excited state, with emission bands occurring at for the deactivation of ^5^D_4_ → ^7^F*_J_* (*J* = 6–3) [*c.f*. Figure 5)]. This emission was clearly visible to the naked eye upon placing the sample under a UV‐lamp both in solution and for the solid state. The ^5^D_4_→^7^F_6_ transition is electrically dipole and is hypersensitive and appears quite strong for both complexes. The ^5^D_4_→^7^F_5_ transition is magnetically dipole in nature and seen here as the most intense transition [57]. This transition exhibited a crystal field splitting (Stark splitting), and is mainly responsible for the green emission, which is viable to the naked eye as stated.

**FIGURE 5:**
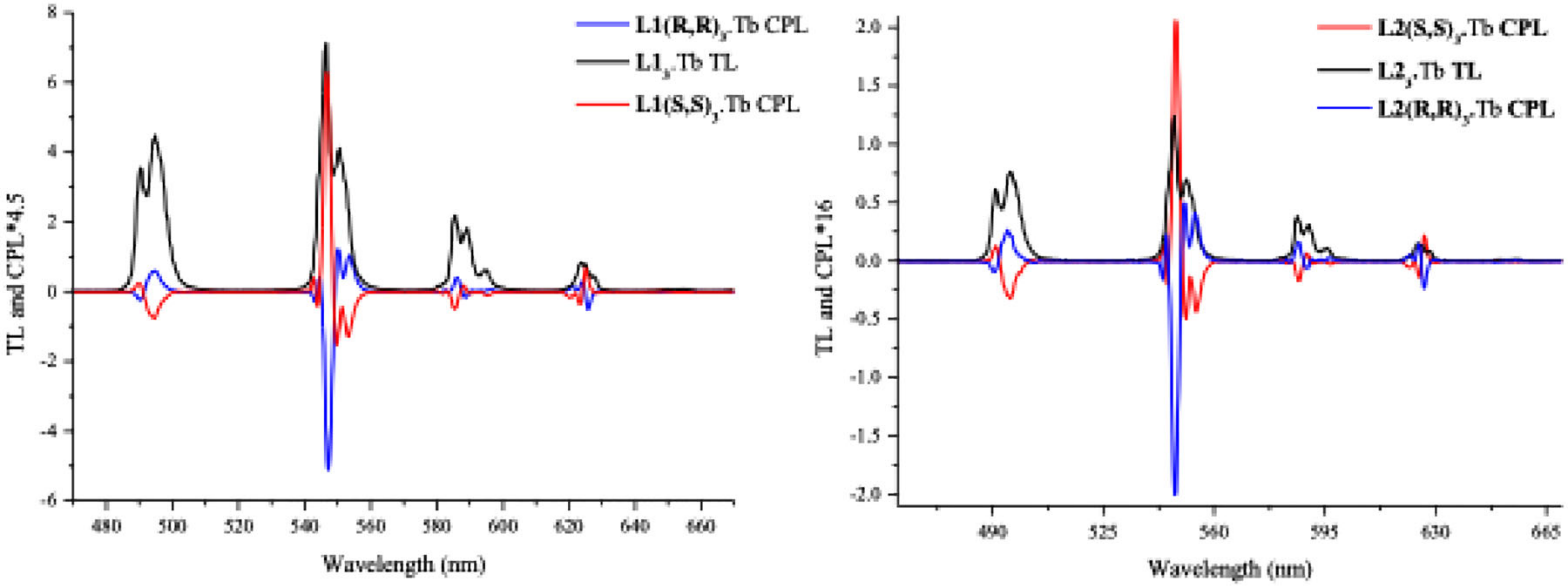
The total luminescence is shown in black for the Tb(III) complexes of **L1** and **L2**. The CPL of both enantiomers are shown; left for the Tb(III) complexes of **L1** and right for **L2**. These are displayed in red color for the *SS* isomers, and in blue for the *RR* isomers. All spectra were recorded in CH_3_CN solution at room temperature upon excitation at 275 nm. The CPL emission bands shown for the four ^5^D_4_ → ^7^F*_J_* (*J* = 6–3) transitions occurring from left to right, respectively.

Excited state Tb(III) lifetime measurements were performed in MeOH and MeOD, upon excitation at the 275 nm, and by monitoring the Tb(III) most intense emission transitions. The decays were best fitted to mono‐exponential decay, resulting in lifetimes of 1.703 and 2.086 ms, respectively. From these results a hydration state of 0.65 was obtained (See Table in Supporting Information). Likewise, the Tb(III) emission lifetimes of the *R,R* isomer (**L1a**) were measured as 1.693 and 2.008 ms, indicating a hydration state of 0.53 (or a q‐value of ∼0) for [Tb**(L1a)_3_**.](OTf)_3_.

The Tb(III) emission of the **L2** complexes were recorded and showed the successful sensitization of the Tb(III) ^5^D_4_ excited state (See Figure 5, right). The Tb(III) emission from **L1** and **L2** appear identical, indicating minimal or no effect by the conversion of the methoxy group to the corresponding hydroxy group. As for **L1**, the hypersensitive ^5^D_4_→^7^F_6_ transition of **L2** appears strong while the ^5^D_4_→^7^F_5_ transition is the most intense for these set of complexes. The Tb(III) lifetimes were also recorded, but from these measurements it was clear that the hydroxy groups affected the excited state lifetimes indicating possible hydrogen bonding interactions with the solvent which gave rise to *q*‐values that did not reflect the true nature of the coordination environment of the complex, as the binding constants were determined to be almost the same as for **L1** (See Tables in ESI). To investigate this further, and to rule out that the above lifetime measurements were not due to dissociation of the **L2** Tb(III) complexes, we also recorded the circular polarized luminescence (CPL) of all four complexes (**L1** and **L2** complexes).

### 2.4 CPL Studies of Tb(III) Complexes of **L1** and **L2**

As was demonstrated above, in Figure 1, both ligands gave rise to CD spectra that were mirror images. Having formed the complexes, the CPL was recorded of both complexes. Figure 5 shows both the total luminescent and the CPL spectra recorded, all four complexes, generated from the two ligand types. Here, each of the characteristic Tb(III) transitions were clearly visible that is ^5^D_4_ → ^7^F*_J_* (*J* = 6–3). Furthermore, as above, the CPL form the two set of complexes appeared to identical; further supporting the observation above, that the *q*‐values for **L2** were not due to dissociation of the ligand from Tb(III). The two pairs of enantiomers showed signals of equal magnitude and opposite signs for the two sets of complexes, demonstrating their enantiomeric relationship. Both sets of complexes also gave rise to same signs (positive or negative) for the respective stereoisomers, demonstrating spectral uniformity for sets of complexes. Hence, both **L1b** and **L2b** (the *R,R* isomers) showed the same signs, while the **L1a** and **L2a** (the *S,S* isomers) displayed the opposite signs for the same *J* transitions (see Supporting Information for plot of *g*_lum_ *vs* wavelength for **L1**). From this analysis, the ^5^D_4_→^7^F_5_ and the ^5^D_4_→^7^F_3_ transitions when measured at 547.1 and 625.3 nm, receptively, give rise to the largest *g*_lum_ values for these complexes of *ca*. ±0.5 (for mirroring transitions). This would also indicate that the removal of the methyl ether in **L2** does not change the chiral nature of these complexes in comparison to **L1**. This was confirmed in the CD spectra for the ligands above (See also discussion below).

By comparing these results with those obtained previously in our laboratory, and by examining the solid‐state structures displayed in Figure 4, it is possible to determine the handedness at the Tb(III) centers in these complexes as either Δ or Λ. The results showing that the *S,S* stereochemistry of the ligand (i.e. **L1b** and **L2b**) results in the formation of Δ stereochemistry around the Tb(III) ion, while the *R,R* (i.e. **L1a** and **L2a**) results in the opposite Λ stereochemistry. This agrees with what we have observed before. We were also able to demine *g*_lum_ for these complexes, at different transitions as shown in Figure 5. Analysis of these data showed that the *g*_lum_ values for the ^5^D_4_→^7^F_5_ were quite large, being measured as (equal and opposite signs) ∼0.45 and ∼0.35 for the ^5^D_4_→^7^F_3_ transition, respectively, in CH_3_CN solutions. These values are similar in magnitude to that often seen for Eu(III) complexes, demonstrating that these Tb(III) complexes also give rise to relatively strong CPL even if simple anisole antenna is employed.

### 2.5 Spectroscopic Absorption and Emission Studies of Tb(III) Complexes of **L1** and **L2** Under Kinetic Control at Room Temperature

Having examined the photophysical properties of the isolated complexes, we next investigated their self‐assembly properties with Tb(III) in solution at room temperature, monitoring the changes in the absorption, Tb(III) emission and CD spectra. All self‐assembly studies were performed at 1x10^−5^ M (ligand) and titrated with Tb(OTf)_3_ (6.6x10^−4^ M) in spectroscopic grade MeCN.

The changes in the ground state for **L1b** are shown in Figure 6 (See Supporting Information for **L1a** and **L1b**). Here, both the absorption bands associated with the pyridine and anisole moieties experience a hyperchromic effect upon the addition of Tb(OTf)_3_, indicating self‐assembly and complex formation between the ligands and Tb(III) ion. Additionally, a small blue shift of the absorption band at approximately 225 nm, as well as a broadening of the spectral absorbance from 200–225 nm in this region up to 1eq of added Tb(III). After this, only minor changes are observed in the absorption spectrum.

**FIGURE 6:**
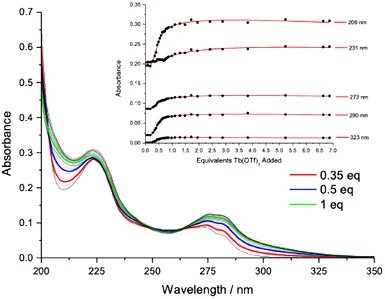
The global changes in the absorption spectra of **L1a** (10 µM) upon titration with Tb(III) (triflate solution). Insert: The changes in various wavelengths as a function of added equivalents of Tb(III), and the corresponding non‐linear regression analysis fit of these.

Changes in the absorption spectrum were analyzed by fitting the global changes by using the non‐linear regression analysis programme SPECFIT (the fit to the data at different wavelengths is shown as insert in Figure 6 for the titration of **L1a** in Figure 7). The results for both enantiomers, showed that the best fit is obtained when the data is fitted to the presence of 3 species, the 3:1, 2:1 and 1:1 (**L:Tb**). From these fits, we were able to determine the stepwise binding constants for the formation of these stoichiometries in solution. Expressed as logβ_L:Tb_, these were shown to be: logβ_1:1_ = 6.45 (±0.16), logβ_1:2_ = 13.01 (±0.27) and logβ_1:3_ = 18.36 (±0.46) for **L1a** (These values summarized for both ligands systems in Table in ESI). These values are comparable to those obtained for the naphthalene analogues of this structure, though overall they are slightly lower.

**FIGURE 7:**
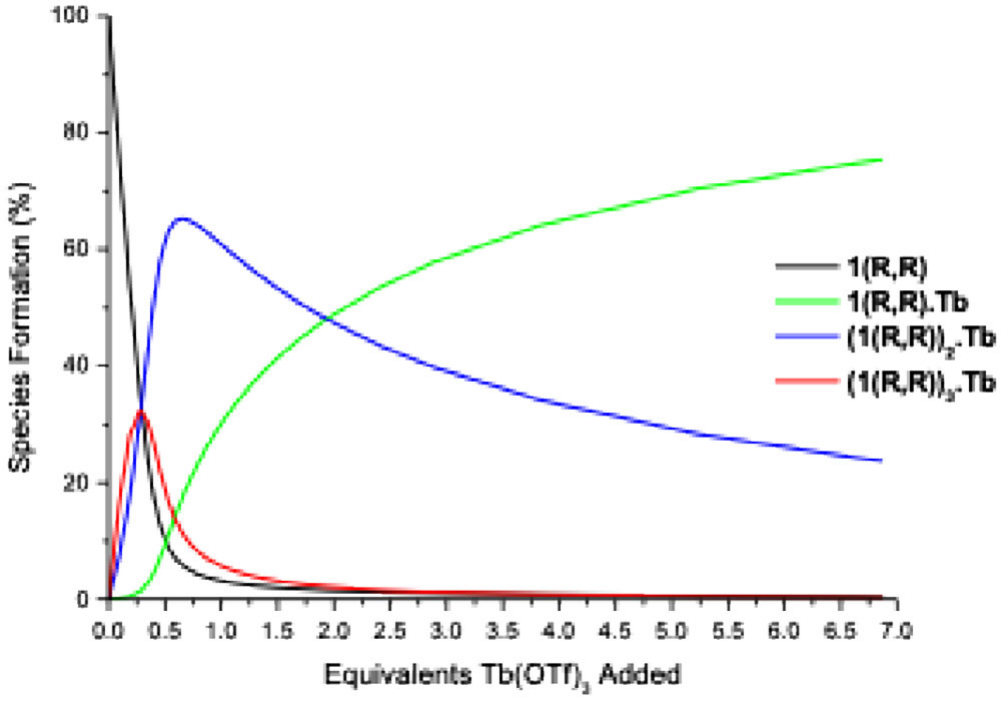
The speciation distribution diagram for the titration shown in Figure 6 for **L1a** with Tb(III).

The speciation distribution diagram obtained from the ground state changes for the titration of **L1a** is shown in Figure 7, demonstrating that upon addition of 0.3 equivalents of Tb(III), the desired 3:1 assembly is formed in *ca*. 30% yield, with the two other main species being that of the ligand **L1b** and the formation of the 2:1 stoichiometry. This is a somewhat lower yield than is seen for other analogues of this ligand at room temperature, such as those having naphthalene units, which normally give rise to 50–70% yield of the 3:1 stoichiometry in solution. Further additions of Tb(III) result in the formation of the 2:1 and the eventual 1:1 stoichiometries (See Supporting Information for the titration isotherms for **L1** obtained from the changed in the UV–vis absorption spectra).

Similar results were obtained for the titration of the *S,S* isomer **L1b** with Tb(III) (see Supporting Information). Analysis of the changes in the absorption spectra, however, demonstrated that the desired 3:1 self‐assembly was formed in *ca*. 45% yield after the addition of 0.33 equivalents of Tb(III) (see Supporting Information). However, it also must be pointed out that this ‘in situ yield’ is determined from the changes in the absorption spectra (see Supporting Information), which is normally, less pronounced than that seen in the excited states (either S_1_ or T_1_). Fitting of the changes in the absorption spectra showed similar binding constants to that of **L1b**, with logβ_1:1_ = 6.63 (±0.26), logβ_1:2_ = 13.38 (±0.41) and logβ_1:3_ = 18.49 (±0.66).

Changes in the evolution of the Tb(III) emission (by monitoring the changes in the phosphorescence emission spectrum) which is ‘switched on’ upon excitation of the ligand was monitored. The global change observed in the Tb(III) emission upon titration of **L1a** are shown in Figure 8a. Enhancement in Tb(III) centered emission increased up to the addition of *ca*. 0.75 equivalents of Tb(III). Thereafter, the emission is quenched which is normally due to dissociation of the initial self‐assembly products formed in situ, into other stoichiometries. To assess this, the changes in the Tb(III) emission were fitted using non‐linear regression analysis, programme SPECFIT, the fitting at the various wavelengths (Figure 8b) demonstrating a good fit to the experimental data.

**FIGURE 8:**
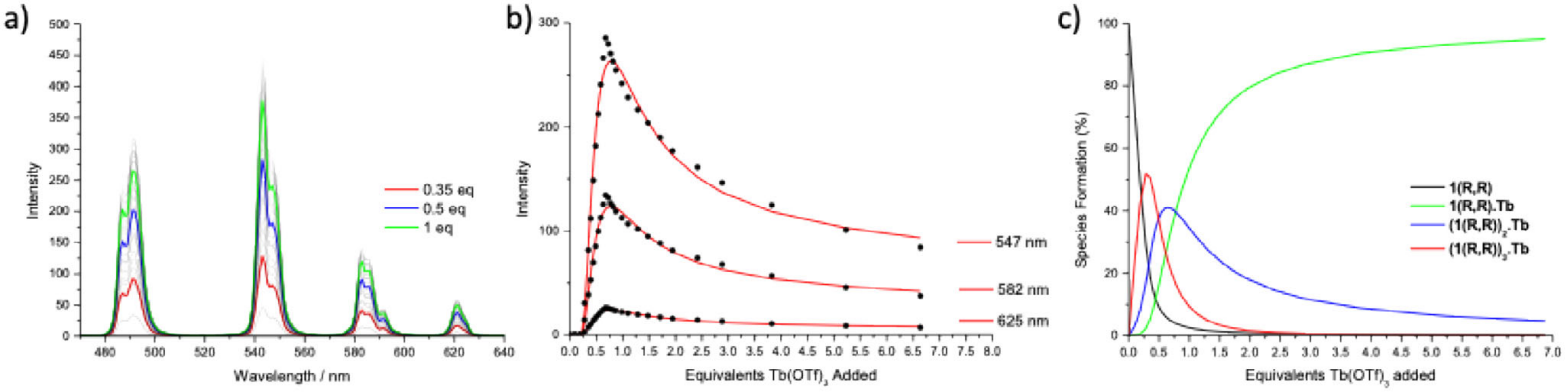
(a) The global changes in the Tb(III) emission spectra upon titrating **L1a** with Tb(III) (triflate solution). (b) The changes in various wavelengths as a function of added equivalents of Tb(III), and the corresponding non‐linear regression analysis fit (in red color) of these. (c) The speciation distribution diagram obtained for the fitting of the changes observed in the Tb(III) emission for the titration of **L1a** with Tb(III).

The speciation distribution for the changes in the phosphorescence emission is shown in Figure 8c and shows that the desired 1:3 stoichiometry is formed in over 50% yield at 0.3 equivalents of Tb(III), while the 1:2 stoichiometry exist in *ca*. 25% yield at the same equivalent. At higher equivalates of Tb(III), the 1:1 stoichiometry becomes the main species in solution, while the 1:2 structure is also dominant; the trend here being like that found in the ground state. The binding constants were calculated from the fitting as: logβ_1:1_ = 6.96 (±0.13), logβ_1:2_ = 13.11 (±0.20) and logβ_1:3_ = 19.01 (±0.34) (See Table S1). These are slightly higher, but within experimental error, of that seen in the fitting of the changes in the absorption spectra.

Likewise, the changes in the evolving Tb(III) emissions for the titration of **L1b** with Tb(III) were monitored (See Supporting Information for full titrations for both ligands). As demonstrated for **L1a** the Tb(III) emission was ‘switched on’ and a good fit was observed for the non‐linear regression analysis of the phosphorescence emission. The global data was fitted in the same manner as described above and gave comparable results (see SI), though the value for the formation of the 1:3 stoichiometry was somewhat lower, being more in line with that seen in the absorption spectra. These values are also summarized in Table S1 (See Supporting Information).

Having carried out the above investigation, we extended this work to analyze the same spectroscopic changes in the **L2** ligands upon titration with Tb(III). The global changes in the absorption spectra for **L2a** are summarized in Figure 9, as well as the changes in the Tb(III) emission, and the corresponding non‐linear regression analysis of the Tb(III) centered emission (See Supporting Information for full titrations for both ligands). As can be seen from these changes, they are overall, very similar to that seen for ligand **L1**, again demonstrating, that the removal of the methoxy ether to yield the hydroxy analogue in **L2** does not have a significant effect on the spectroscopic changes in any significant manner. Analysis of the fitted data, showed that the binding constants were comparable for both the absorption and the changes in the Tb(III) emission, being logβ_1:1_ = 7.06 (±0.11), logβ_1:2_ = 13.21 (±0.17) and logβ_1:3_ = 19.11 (±0.29) (Table S1) for the titration of **L2a** with Tb(III) upon analysis the changes in the Tb(III) emission. Similar spectral and non‐linear regression analysis results were also seen for **L2b** (See Supporting Information and Table S1).

**FIGURE 9:**
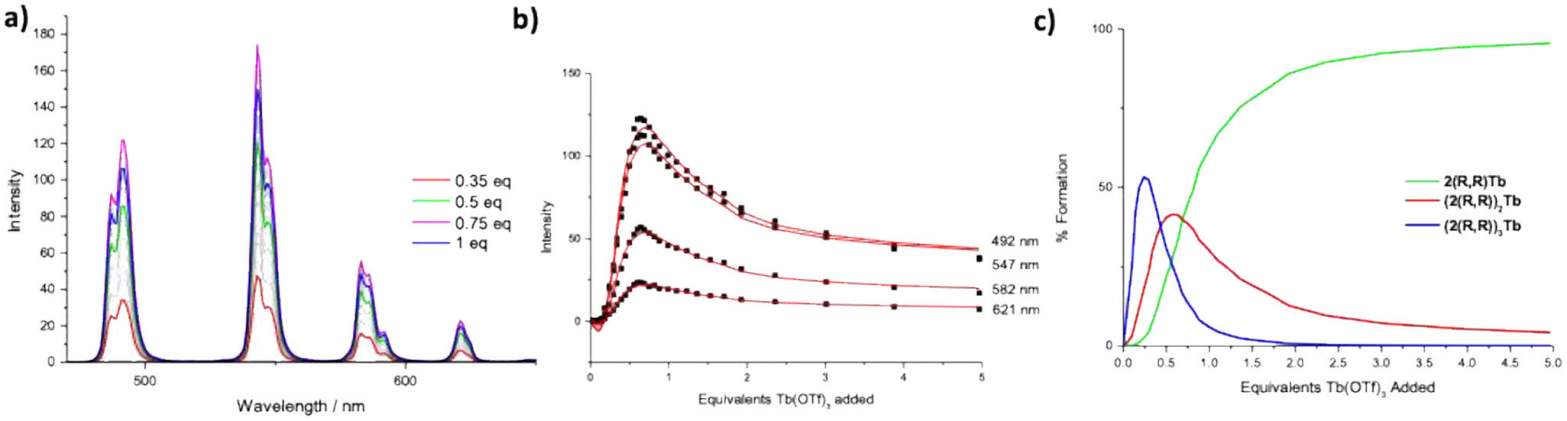
(a) The global changes in the absorption spectra **L2a** with Tb(III) (triflate solution). (b) The corresponding global changes in the Tb(III) centred emission (recorded in phosphorescence mode). (c) The changes in various Tb(III) transitions as a function of added equivalents of Tb(III), and the corresponding non‐linear regression analysis fit of these (in red colour), which showed good fit to the experimental data (the ligand was omitted from the graph in this analysis).

### 2.6 Circular Dichroism Studies of Tb(III) Complexes of **L1** and **L2** Under Kinetic Control at Room Temperature

As was clear from the discussion above, all four complexes gave rise to CPL signals when the Tb(III) emission was recorded. Furthermore, the CD spectra of the two sets of ligands also gave rise to mirror‐image dichroism bands for both sets of ligands. Because of this, we also measured the CD spectra of all four ligands in CH_3_CN at room temperature upon addition of 0 (i.e. free ligand) 0.33, 0.5 and 1.0 equivalents of Tb(III), these representing the three stoichiometries determined from the speciation distribution diagrams above. The results are shown in Figure 10 demonstrating major changes in all the bands. As was the case in the CPL, the two sets of ligands show same overall trends in their spectra as a function of Tb(III) indicating that the removal of the methyl ether does not change their overall chiral signatures. In fact, from the changes in Figure 10, individual chiral ‘stochiometric’ signatures can be identified; that is to say, each of the three spectra assigned to the 3:1, 2:1 and 1:1 (**L:Tb**) species is clearly identifiable in Figure 10. These same signatures can be seen also in Figure 10b for the changes observed in **L2**. As an example, in the case of **L1a**, the two prominent negative dichroism bands ‘enhance in intensity’, where the high energy band centered at 230 nm, previously displaying a negative Cotton effect, becomes more ‘positive’ after the formation of the 3:1 complex, and a new band, that had a slightly positive Cotton effect at *ca*. 240 nm for the ligand, now becomes a signal with a negative Cotton effect. Similarly, the long wavelength band centered at *ca*. 278 nm is slightly enhanced. The same effect is then mirrored for the changes seen in **L1b**. The ‘sinusoidal‐like shapes’ of the CD spectra for the complexes vs. those of the ligands, might suggest exciton coupling between the antennae, being due to the Tb(III) ion, which brings these chromophores/antennae into proximity as discussed above.

**FIGURE 10:**
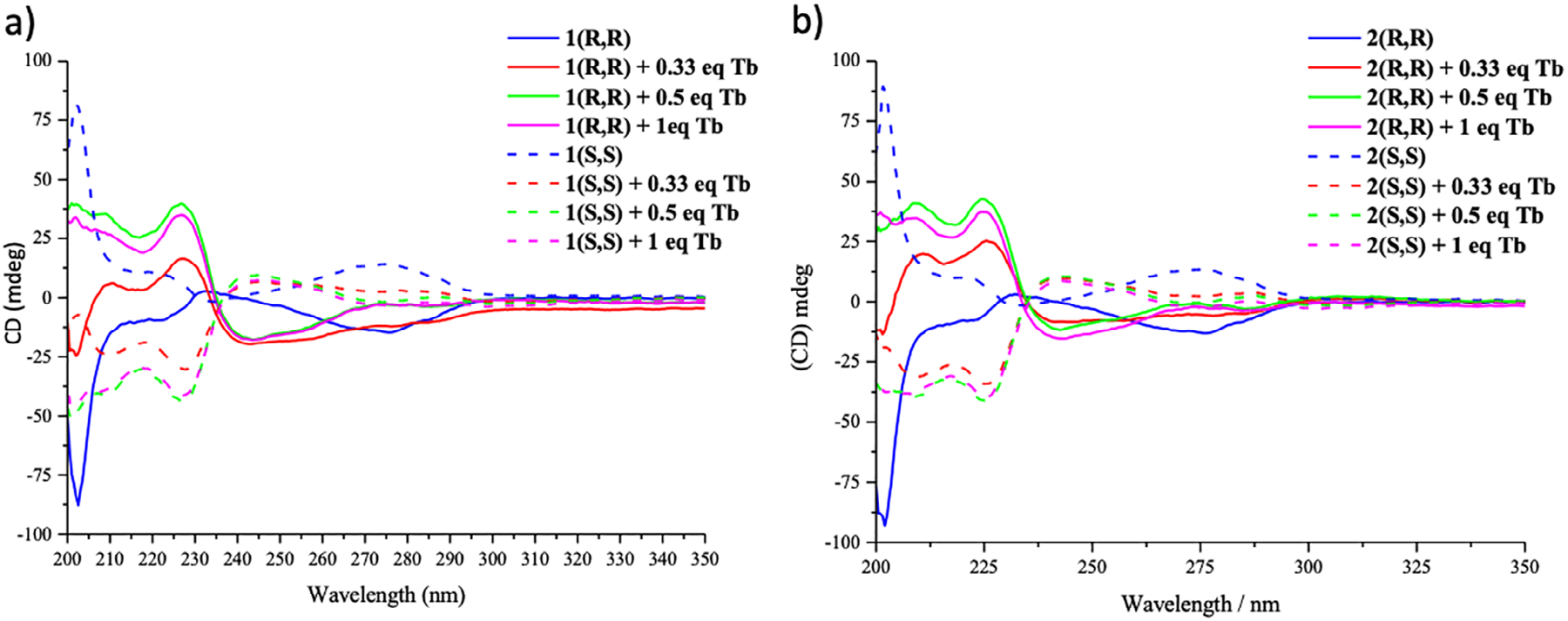
The changes in the CD spectra of: (a) **L1a** and **L1b** (2 mM) and (b) **L2a** and **L2b** (2 mM) upon addition of Tb(III), showing the dramatic changes occurring upon the self‐assembly formation in CH_3_CN at room temperature at 0.33, 0.5, and 1 equivalents of Tb(III).

## 3 Conclusion

In conclusion, we have developed four chiral ligands, possessing two stereogenic centers each based on the **pda** design. Of these, **L1** were based on the use of 4‐methoxyphenyl antennae, the methoxy group of which was cleaved using BBr_3_, to yield the phenol analogues (**L2**). Using Tb(III) we formed complexes of all using microwave assisted synthesis; these being formed in 3:1 (**L:Tb**) stoichiometries. The crystal structure of the Tb(III) complex of **L1b** confirmed the structural nature of this in the solid state. All complexes possess rich photophysical properties, some of which we probed in this investigation. From the ground state changes, it was clear that the interaction with Tb(III) gave rise to coordination of the ligand which resulted in hyperchromism. The complexes gave rise to Tb(III) centered emission, upon excitation of the organic antennae. The complexes had Tb(III) excited state lifetimes measured in MeOH which were of similar magnitudes to those seen for related complexes. The complexes also gave rise to mirror image CPL spectra, while the CD allowed for identification of three distinct stoichiometries in solution when investigated under kinetic control. We also investigated the self‐assembly of these ligands with Tb(III) at room temperature in CH_3_CN solution with the view of determining the binding constants for these equilibria. Non‐linear regression analysis of both the changes in the ground and the Tb(III) excited state (recoded in phosphorescence mode) showed that under kinetic conditions, the ‘desired’ 3:1 stoichiometry was formed in *ca*. 40%–50% yields upon the addition of 0.33 equivalents of Tb(III) at room temperature. While also the 2:1 and the 1:1 species were present; the latter becoming dominant at higher Tb(III) equivalents (*c.f*. values in Supporting Information Table S2). Likewise, (as mentioned above) the changes in the CD demonstrated that the ligands **L1** and **L2** underwent similar self‐assembly formation with Tb(III) under kinetic control as the changes were close to identical for both sets. The results seem to indicate that the substitution in this case on the antenna does not interfere with the overall structure of the self‐assembly.

The results presented herein are an important contribution to the field of lanthanide directed self‐assembly formation from chiral ligands, as the phenolic moiety on these ligands allows for further functionalization, such as introduction of long chains possessing additional functionalities, at the antennae sites that would facilitate the formation of higher order systems and polymers from such ligands, while preserving the integrity of both the structure and the photophysical properties of the assembly. We are currently investigating these features in greater detail.

## Conflicts of Interest

The authors declare no conflicts of interest.

## Supporting information



The authors have cited additional references within the Supporting Information (SI). Crystallographic data have been deposited with the CCDC under deposition numbers CCDC 2490133, 2490134, 2491642, and 2491643. This can be obtained via https://www.ccdc.cam.ac.uk/structures. **Supporting File 1**: chem71535‐sup‐0001‐SuppMat.docx

## Data Availability

The data that support the findings of this study are available from the corresponding author upon reasonable request.
