# Supplementary material for: Lanthanide Centered and Circular Polarized Luminescence (CPL) From Tb(III) Complexes Made From Structurally Simple Methoxy‐Phenyl and Phenol‐Derived Pyridyldiamide (pda) Ligands
Source: Chemistry. 2026 Aug 11;32(41):e71535. doi: 10.1002/chem.71535 (PMC13729993; doi:10.1002/chem.71535)
Supplement: Supplementary file 1 [file CHEM-32-e71535-s001.docx]

**Lanthanide centred and circular polarized luminescence (CPL) from Tb(III) complexes made from structurally simple methoxy-phenyl and phenol derived pyridyldiamide (pda) ligands**

Jennifer E. Jones,^[a]^ Jonathan A. Kitchen,*^[b]^ Robert Peacock,^[c]^ Thorfinnur Gunnlaugsson*^[a,b]^

**Supporting Information (SI)**

Experimental

Materials and methods

All solvents and chemicals/reagents were purchased from commercial sources and used without further purification.

Synthesis

Synthesis of Ligands L1a(R,R), L1b(S,S), L2(R,R) and L2(S,S) L1a(*R,R*):

L1a: (*R,R*)-*N*,*N’*-Bis(1-(4-methoxyphenyl)ethyl)pyridine-2,6-dicarboxamide

(*R*)-(+)-1-(4-methoxyphenyl)-ethylamine (2.88 mL, 19.50 mmol), triethyl amine (5.40 mL, 38.74 mmol), 2,6-dipyridine carboxylic acid (1.60g, 9.58 mmol) and HOBt (5.68 mg, 42.04 mmol) were dissolved in dichloromethane (300 mL) and stirred at 0 °C for 15 minutes under inert conditions. EDCI.HCl (7.34 g, 38.29 mmol) was added and the solution continued stirring at 0 °C for 30 minutes, then for a further 36 hours at room temperature. The organic solution was washed with NaHCO_3_ (sat) (3 x 25 mL), deionised water (2 x 30 mL), brine (1 x 30 mL) and the organic fractions combined and dried over MgSO_4_, filtered and then evaporated to dryness under reduced pressure. The white residue was precipitated from a concentrated acetonitrile solution and isolated as a white powder (2.224 g, 54 %). MP: 173-174 °C. ^1^H NMR (600 MHz, CDCl_3_) δ_H_: 8.35 (d, 2H, *J* = 7.5 Hz, pyr-*H*), 8.03 (t, 1H, *J* = 7.9 Hz, pyr-*H*), 7.75 (d, 2H, *J* = 7.9 Hz, N*H*), 7.34-7.31 (m, 4H, Ar-*H*), 6.94-6.90 (m, 4H, Ar-*H*), 5.30-5.24 (m, 2H, C*H*), 3.83 (s, 6H, -OC*H*_3_), 1.60 (s, 3H, -C*H*_3_), 1.59 (s, 3H, -C*H*_3_). ^13^C{^1^H} (150 MHz, CDCl_3_) δ_C_: 162.4, 159.1, 148.8, 139.0, 135.0, 127.3, 125.0, 114.1, 55.3, 48.5, 21.7 ppm. IR (solid) ν: 3277, 3080, 2974, 2930, 2833, 1672, 1641, 1614, 1587, 1568, 1511, 1442, 1336, 1325, 1308, 1278, 1247, 1215, 1205, 1177, 1167, 1124, 1104, 1078, 1034, 1009, 999, 934, 903, 870, 829, 810, 733, 674, 645, 608, 580, 553 cm^-1^. λ_max_ (ε / mol^-1^ dm^3^ cm^-1^): 224 (31,449), 275 (9673). HRMS ESI^+^ calculated for C_25_H_27_N_3_O_4_Na: [L+Na]^+^ *m/z* = 456.1899; found 456.1904. Elemental analysis for C_25_H_27_N_3_O_4_ calc: C 69.27, H 6.28, N 9.69; found: C 69.02, H 6.19, N 9.67 %.

**L1b: (S,S)-*N*,*N’*-Bis(1-(4-methoxyphenyl)ethyl)-pyridine-2,6-dicarboxamide**

The *S,S*-enantiomer was obtained *via* the same experimental methodology as reported for the *R,R*-enantiomer using: (S)-(-)-1-(4-methoxyphenyl)-ethylamine (2.88mL, 19.50 mmol); triethyl amine (5.40 mL, 38.74 mmol); 2,6-dipyridine carboxylic acid (1.60g, 9.58 mmol); HOBt (5.68 mg, 42.04 mmol); EDCI.HCl (7.34 g, 38.29 mmol) and dichloromethane (300 mL) affording the title compound as a white powder (2.005g, 48 %). MP: 173-175 °C. ^1^H NMR (600 MHz, CDCl_3_) δ_H_: 8.34 (d, 2H, *J* = 8.1 Hz, pyr-*H*), 8.02 (t, 1H, *J* = 8.1 Hz, pyr-*H*), 7.79 (d, 2H, *J* = 8.1 Hz, N*H*), 7.33-7.30 (m, 4H, Ar-*H*), 6.92-6.89 (m, 4H, Ar-*H*), 5.30-5.22 (m, 2H, -C*H*), 3.82 (s, 6H, -OC*H*_3_), 1.59 (s, 3H, -C*H*_3_), 1.58 (s, 3H, -C*H*_3_) ppm. ^13^C{^1^H} (150 MHz, CDCl_3_) δ_C_: 162.6, 159.2, 149.0, 139.2, 135.2, 127.5, 125.2, 114.3, 55.5, 48.7, 21.9 ppm. IR (solid) ν: 3274, 3079, 2974, 2931, 2833, 1672, 1641, 1614, 1589, 1512, 1433, 1376, 1326, 1309, 1288, 1248, 1178, 1124, 1105, 1078, 1035, 1009, 1000, 934, 904, 870, 830, 810, 734, 693, 675, 645, 626, 616, 585, 579, 570, 555 cm^-1^. λ_max_ (ε / mol^-1^ dm^3^ cm^-1^): 224 (32,694), 275 (10,091). HRMS ESI^+^ calculated for C_25_H_27_N_3_O_4_Na: [L+Na]^+^ *m/z* = 456.1899; found 456.1884. Elemental analysis for C_25_H_27_N_3_O_4_ calc: C 69.27, H 6.28, N 9.69; found: C 69.12, H 6.25, N 9.72 %.

**L2a:** **(*R,R*)-*N*,*N’*-bis(1-(4-hydroxyphenyl)ethyl)pyridine-2,6-dicarboxamide**

In a thoroughly dried flask, (R,R)-*N*,*N’*-bis(1-(4-methoxyphenyl)ethyl)pyridine-2,6-dicarboxamide (1.00 g, 2.3 mmol) was dissolved in dry dichloromethane (100 mL) under inert conditions and the solution cooled to 0 °C. Boron tribromide (2 mL, excess) was added drop-wise to the solution over a period of 5 minutes and stirring continued at 0 °C for a further 15 minutes, after which time additional boron tribromide (1 mL) was added and the ice bath removed. The dark orange solution was stirred at room temperature for 12 hours, during which time the solution turned yellow. The reaction was cooled to 0 °C and deionised water (15 mL) added slowly to quench the reaction. The reaction mixture was neutralised to pH 7 with NaHCO_3_ (sat.) solution and the organic solvent removed under reduced pressure. The aqueous solution was the extracted with ethyl acetate (2 x 20 mL), the organic fractions combined and washed with deionised water (1 x 20 mL), brine (1 x 20 mL), dried over MgSO_4_, filtered and then evaporated to dryness under reduced pressure. The residue was taken up in the minimum volume of acetonitrile and stirred at ambient temperature until a precipitate formed which was collected by filtration to afford the title compounds as a white powder (0.558g, 60%). Melting point: 222-224 °C. ^1^H NMR (400 MHz, CD_3_OD) δ_H_: 8.24 (d, 2H, *J* = 7.9 Hz, pyr-*H*), 8.10 (m, 1H, pyr-*H*), 7.27-7.24 (m, 4H, Ar-*H*), 6.78-6.75 (m, 4H, Ar-*H*), 5.24 (q, 2H, *J* = 7.3 Hz, C*H*), 1.58 (s, 3H, -C*H*_3_), 1.56 (s, 3H, -C*H*_3_) ppm. ^13^C{^1^H} (100 MHz, CD_3_OD) δ_C_: 164.2, 156.6, 149.8, 139.3, 134.6, 127.5, 125.1, 115.2, 48.7, 20.7 ppm. IR (solid) ν: 3402, 3384, 3299, 3093, 3025, 2967, 2951, 2926, 2867, 1668, 1638, 1615, 1598, 1568, 1522, 1445, 1379, 1359, 1335, 1282, 1261, 1217, 1173, 1131, 1102, 1080, 1002, 937, 917, 861, 840, 829, 795, 759, 745, 721, 663, 642, 599, 573 cm^-1^. UV-vis. λ_max_ (ε / mol^-1^ dm^3^ cm^-1^): 222 (27,341), 275 (9571). HRMS ESI^+^ calculated for C_23_H_23_N_3_O_4_Na: [L+Na]^+^ *m/z* = 428.1586; found 428.1586. Elemental analysis for C_23_H_23_N_3_O_4_.0.5H_2_O calc: C 66.65, H 5.84, N 10.14; found: C 66.98, H 5.81, N 10.54 %.

The ^1^H NMR (400 MHz recorded in (CD_3_)_2_SO) is also given for comparison below, being identical to that seen for **L2b**.

**L2b: (S,S)-*N*,*N’*-bis(1-(4-hydroxyphenyl)ethyl)pyridine-2,6-dicarboxamide**

The *S,S*-enantiomer was obtained *via* the same experimental methodology as reported for the *R,R*-enantiomer using: (S,S)-*N*,*N’*-bis(1-(4-methoxyphenyl)ethyl)pyridine-2,6-dicarboxamide (1.00 g, 2.3 mmol); boron tribromide (2 mL, excess) and dry dichloromethane (100 mL) affording the title compound as a white powder (0.523 g, 56 %). MP: 223-224°C. ^1^H NMR (400 MHz, (CD_3_)_2_SO) δ_H_: 9.24 (d, 2H, *J* = 8.8 Hz, Pyr-*H*), 8.21-8.13 (m, 3H, 2x-N*H*, Pyr *H*), 7.25-7.21 (m, 4H, Ar*H*), 6.75-6.71 (m, 4H, Ar*H*), 5.22-5.12 (m, 2H, C*H*), 1.55 (S, 3H, -C*H*_3_), 1.53 (s, 3H, -C*H*_3_) ppm. ^13^C{^1^H} (100 MHz, (CD_3_)_2_SO) δ_C_: 162.6, 156.5, 149.2, 139.4, 133.9, 127.3, 124.6, 115.0, 45.5, 21.8 ppm. IR (solid) ν: 3385, 3306, 3024, 2966, 2951, 2924, 2867, 1668, 1634, 1615, 1597, 1568, 1516, 1445, 1379, 1359, 1334, 1282, 1261, 1218, 1173, 1130, 1102, 1090, 996, 936, 916, 860, 840, 829, 759, 745, 721, 663, 641, 616, 599, 573 cm^-1^. UV-vis. λ_max_ (ε / mol^-1^ dm^3^ cm^-1^): 222 (23,705), 275 (8269). HRMS ESI^-^ calculated for C_23_H_22_N_3_O_4_: [L-H]^-^ *m/z* = 404.1610; found 404.1595.

**Synthesis of Tb(III) complexes of L1a, L1b, L2a and L2b**

**General procedure:** An oven dried microwave vial was charged with the appropriate ligand (3 equiv.), Tb(CF_3_SO_3_)_3_ (1 equiv.), dissolved in MeOH (5 mL), sealed and heated at 70 °C under microwave irradiation for 20 minutes. The resulting colourless solutions were subjected to slow vapour diffusion of diethyl ether at ambient temperature resulting in white crystalline powders.

**[(L1a(*R,R*))_3_Tb.(OTf)_3_]:** **L1a** (30 mg, 0.07 mmol), Tb(CF_3_SO_3_)_3_ (14.1 mg, 0.023 mmol) afforded [(**1**(*R,R*))_3_Tb.(OTf)_3_] as a white crystalline solid (23.1 mg, 53%). δ_H_ (CD_3_OD, 400 MHz): 37.19, 34.18, 20.86, 17.42, 12.60, -14.92 ppm. IR (solid) ν: 3281, 3095, 2978, 2938, 2840, 1627, 1593, 1559, 1513, 1459, 1383, 1364, 1342, 1282, 1225, 1176, 1162, 1127, 1106 1028, 952, 918, 867, 831, 812, 756, 672, 660, 635, 595, 572, 556 cm^-1^. Elemental analysis for C_78_H_81_F_9_N_9_O_21_S_3_Tb.2H_2_O calc: C 48.22, H 4.4.1, N 6.49; found C 48.01, H 3.58, N 5.98 %.

**[(1(*S,S*))_3_Tb.(CF_3_SO_3_)_3_]:** **L1b** (40 mg, 0.092 mmol), Tb(CF_3_SO_3_)_3_ (18.6 mg, 0.03 mmol) afforded [(**L1b**(S,S))_3_Tb.(CF_3_SO_3_)_3_] as a white crystalline solid (32.9 mg, 45 %). δ_H_ (CD_3_OD, 400 MHz): 37.06, 34.10, 20.80, 17.38, 12.56, -14.88 ppm. IR (solid) ν: 3282, 3096, 2977, 2938, 2840, 1627, 1593, 1559, 1513, 1460, 1383, 1363, 1343, 1283, 1226, 1176, 1161, 1127, 1105, 1028, 952, 917, 867, 831, 812, 756, 672, 661, 635, 599, 594, 586, 572, 554 cm^-1^. Elemental analysis for C_78_H_81_F_9_N_9_O_21_S_3_Tb.2H_2_O calc: C 48.22, H 4.4.1, N 6.49; found C 48.37, H 3.91, N 6.32 %.

**[(L2a(*R,R*))_3_Tb.(CF_3_SO_3_)_3_]:** **L2a** (30 mg, 0.074 mmol), Tb(CF_3_SO_3_)_3_ (14.9 mg, 0.024 mmol) afforded [(**L2a**(*R,R*))_3_Tb.(CF_3_SO_3_)_3_] as a white crystalline solid (26 mg, 58 %). IR (solid) ν: 3297, 3093, 2979, 2938, 1628, 1591, 1555, 1515, 1452, 1382, 1359, 1242, 1169, 1125, 1101, 1028, 971, 917, 853, 833, 753, 721, 635, 608, 585, 575 cm^-1^. Elemental analysis for C_72_H_69_F_9_N_9_O_21_S_3_Tb.(C_2_H_5_)_2_O (1895.3611 gmol^-1^) calc: C 48.13, H 4.20, N 6.65; found C 48.22, H 3.55, N 6.55 %.

**[(L2b(*S,S*))_3_Tb.(CF_3_SO_3_)_3_]: L2b** (30 mg, 0.074 mmol), Tb(CF_3_SO_3_)_3_ (14.9 mg, 0.024 mmol) afforded [(**L2b**(*S,S*))_3_Tb.(CF_3_SO_3_)_3_] as a white crystalline solid (13.6 mg, 29 %).

**Figures**

Figure S1: The ^1^H NMR (600 MHz) of ligand **L1a [1(R,R)]** in CDCl_3_

Figure S2: The ^13^C NMR of ligand **L1a [1(R,R)]** in CDCl_3_

Figure S3: The ^1^H NMR (600 MHz) of ligand **L1b [1(S,S)]** in CDCl_3_

Figure S4: The ^13^C NMR of ligand **L1b [1(S,S)]** in CDCl_3_

Figure S5: The ^1^H NMR of ligand **L2a [2(R,R)]** in DMSO-*d_6_*

Figure S6: The ^13^C NMR of ligand **L2a [1(R,R)]** in DMSO-*d_6_*

Figure S7: The ^1^H NMR of ligand **L2a [1(R,R)]** in CD_3_OD-D_4_

Figure S8: The ^13^C NMR of ligand **L2a [1(R,R)]** in CD_3_OD-D_4_

Figure S9: The ^1^H NMR of ligand **L2b [2(S,S)]** in DMSO-*d_6_*

Figure S10: The ^13^C NMR of ligand **L2b** in DMSO-*d_6_*

Figure S11: The CD spectrum of **L2a [L2(R,R)}** in black and **L2b [L2(S,S)]** in red, recorded in spectroscopic grade acetonitrile at 2x10^-3^ moldm^-3^ at room temperature.

Figure S12: The ^1^H NMR (400 MHz) of the 3:1 **1La**:Tb(III) complex (top) and the **1Lb**:Tb(III) (bottom) in MeOD-*d*_4_.

Figure S13: The green Tb(III) emission observed from the solid of **[Tb(L1b)_3_](CF_3_SO_3_)3·H_2_O** under a 275 nm UV lamp.

Figure S14: Tb structure cis- or “horseshoe” ligand conformation

Figure S15: Tb structure intermediate ligand conformation

Figure S16: Tb structure trans- or s-type ligand conformation

**Formation of Eu(III) complex for crystal structure analysis**

We were unable to grow Tb(III) crystals from **L1a** that were of good enough quality for crystal structure analysis. Consequently, we tried to form crystals using Eu(III), which worked. The structure was resolved and is only presented here for compare it with the Tb(III) complex [Tb(**L1b**)_3_](CF_3_SO_3_)_3_·H_2_O formed using the other enantiomer **L1b.**

Colourless crystals of [Eu(**L1a**)_3_](ClO4)_3_·2PrOH were grown by vapour diffusion of diethyl ether into a mixed ethanol/isopropanol solution. These crystals exhibited the characteristic red Eu(III) luminescence under UV irradiation. [Eu(**L1a**)_3_](ClO4)_3_·2PrOH crystallised in the orthorhombic space group *P*2_1_2_1_2_1_ and contained one complete molecule in the asymmetric unit and two interstitial isopropanol molecules – one of the PrOH molecules exhibited significant disorder and could not be modelled satisfactorily. As such, it was treated using the solvent masking routine implemented in Olex2. The three ligands of **L1a** coordinate to the Eu(III) *via* the pyridyl nitrogen and the two amide carbonyl oxygen atoms to give an overall N_3_O_6_ coordination sphere with a distorted tri-capped trigonal prismatic geometry. Bond lengths are consistent with structures of this type [Eu – O range = 2.382(5) - 2.425(5) Å; Eu – N range = 2.539(5) – 2.559(5) Å]. Chirality from ligand **L1a** is transferred to the metal centre which adopts an Λ-configuration. All three ligands adopt the trans-type configuration when viewed from above as the arms are oriented away from opposing faces of the pyridyl ring. The structure contains significant hydrogen bonding between the amide protons and perchlorate counter anions or interstitial solvent (See table below for a full list of H-bonding interactions). The result is a complex network of interactions creating an H-bonded network.

Figure S17: The molecular structures of the Tb(III) (discussed in the main body of the article) (RIGT) and the Eu(III) complexes (LEFT) formed using **L1** as determined by SCXRD. Left: [Tb(**L1b**)_3_](CF_3_SO_3_)_3_·H_2_O and right [Eu(**L1a**)_3_](ClO4)_3_·2PrOH. Anions, hydrogen atoms and interstitial solvent molecules have been omitted for clarity. Atoms shown as 50% probability ellipsoids: Tb (green), Eu (pink), O (red), N (blue), and C (grey).

Figure S18: The global changes in the absorption spectra of **L1b** (10mM) upon titration with Tb(III) (triflate solution).

|  |  |
| --- | --- |

Figure S19: Experimental binding isotherms for the changes in the ground state spectra upon titrating **L1a (R,R)** (right) and **L1b (S,S)** (left) at 1x10^-5^ moldm^-3^ with Tb(CF_3_SO_3_)_3_ in spectroscopic grade acetonitrile at RT and their corresponding fit by means of SPECFIT (**—**).

Figure S20: The Speciation distribution diagram of **L1b** (10mM) as a function of added equivalents of Tb(III), and the corresponding non-linear regression analysis fit of these.

|  |  |
| --- | --- |

Figure S21: Overall changes in the Tb(III) phosphorescence spectra upon titrating **L1a** (left) and **L1b** (right) at 1x10^-5^ moldm^-3^ with Tb(CF_3_SO_3_)_3_ in spectroscopic grade acetonitrile at RT.

|  |  |
| --- | --- |

Figure S22: Experimental binding isotherms for the changes in the phosphorescence spectra upon titrating **L1a** (left) and **L1b** (right) at 1x10^-5^ moldm^-3^ with Tb(CF_3_SO_3_)_3_ in spectroscopic grade acetonitrile at RT and their corresponding fit by means of SPECFIT (**—**).

Figure S23: Overall changes in the UV-vis (ground state) spectra upon titrating **L2b** at 1x10^-5^ moldm^-3^ with Tb(CF_3_SO_3_)_3_ in spectroscopic grade acetonitrile at RT.

|  |  |
| --- | --- |

Figure S24: Experimental binding isotherms for the changes in the ground state spectra upon titrating **L2a** (left) and **L2b** (right) at 1x10^-5^ moldm^-3^ with Tb(CF_3_SO_3_)_3_ in spectroscopic grade acetonitrile at RT and their corresponding fit by means of SPECFIT (**—**)

|  |  |
| --- | --- |

Figure S25: Overall changes in the Tb(III) phosphorescence spectra upon titrating **L2a** (left) and **L2b** (right) at 1x10^-5^ moldm^-3^ with Tb(CF_3_SO_3_)_3_ in spectroscopic grade acetonitrile at RT.

|  |  |
| --- | --- |

Figure S26: Experimental binding isotherms for the changes in the phosphorescence spectra upon titrating **L2a** (left) and **L2b** (right) at 1x10^-5^ moldm^-3^ with Tb(CF_3_SO_3_)_3_ in spectroscopic grade acetonitrile at RT and their corresponding fit by means of SPECFIT (**—**).

|  |  |
| --- | --- |

Figure S27: Speciation-distribution diagram obtained from the phosphorescence titration of **L2a** (left) and **L2b** (right) at 1x10^-5^ moldm^-3^ with Tb(CF_3_SO_3_)_3_ in spectroscopic grade acetonitrile at RT.

Figure S28: Plot of the g_lum_ *vs.* wavelength for the Tb(III) complexes formed from **L1a** and **L1b**.

Table S1 – Hydrogen bond information for [Tb(**L1b**)_3_](CF_3_SO_3_)_3_·H_2_O

| Hydrogen Bonds for [Tb(**L1b**)_3_](CF_3_SO_3_)_3_·H_2_O**.** | | | | | | |
| --- | --- | --- | --- | --- | --- | --- |
| **D** | **H** | **A** | **d(D-H)/Å** | **d(H-A)/Å** | **d(D-A)/Å** | **D-H-A/°** |
| N1 | H1 | O200^1^ | 0.85 | 2.10 | 2.924(5) | 163.2 |
| N3 | H3 | O301^2^ | 0.86 | 2.13 | 2.933(5) | 153.9 |
| N31 | H31 | O400 | 0.85 | 1.96 | 2.797(5) | 169.3 |
| N33 | H33 | O100 | 0.85 | 2.13 | 2.957(6) | 166.3 |
| N61 | H61 | O202 | 0.85 | 2.22 | 2.960(5) | 145.9 |
| N63 | H63 | O102^3^ | 0.82 | 2.12 | 2.911(6) | 161.7 |
| O400 | H40A | O1^4^ | 0.84 | 2.52 | 3.032(5) | 119.8 |
| O400 | H40A | O61 | 0.84 | 2.28 | 2.971(5) | 140.0 |
| O400 | H40B | O300 | 0.92 | 1.92 | 2.809(5) | 162.3 |

^1^1+X,1+Y,+Z; ^2^1+X,+Y,1+Z; ^3^+X,1+Y,1+Z; ^4^-1+X,+Y,+Z

Table S2 – Hydrogen bond information for [Eu(**L1a**)_3_](ClO_4_)_3_·2PrOH

| Hydrogen Bonds for [Eu(**L1a**)_3_](ClO_4_)_3_·2PrOH | | | |
| --- | --- | --- | --- |
| **D** | **A** | **d(D-A)/Å** | **D-H-A/°** |
| N3 | O13 | 2.923(7) | 162.2 |
| N31 | O21A^1^ | 3.043(16) | 161.4 |
| N33 | O15 | 2.897(10) | 167.0 |
| N63 | O22A | 3.057(17) | 142.3 |
| O100 | O11^2^ | 3.095(10) | 148.1 |

^1^1-X,1/2+Y,3/2-Z; ^2^1/2+X,3/2-Y,2-Z

Table S3 – X-ray crystallographic data summary (Shown on next page)

|  | **L1a** | **L1b** | [Eu(**L1a**)_3_](ClO4)_3_·2PrOH | [Tb(**L1b**)_3_](CF_3_SO_3_)_3_·H_2_O |
| --- | --- | --- | --- | --- |
| CCDC entry | 2490134 | 2491642 | 2490133 | 2491643 |
| Empirical formula | C_25_H_27_N_3_O_4_ | C_25_H_27_N_3_O_4_ | C_81_H_97_Cl_3_EuN_9_O_26_ | C_78_H_83_N_9_O_22_F_9_S_3_Tb |
| Formula weight | 433.49 | 433.49 | 1870.98 | 1924.63 |
| Temperature/K | 117.36 | 100.01 | 100.08 | 162.15 |
| Crystal system | tetragonal | tetragonal | orthorhombic | triclinic |
| Space group | *P*4_1_ | *P*4_3_ | *P*2_1_2_1_2_1_ | *P*1 |
| a/Å | 12.8888(9) | 12.8728(12) | 12.7545(6) | 12.748(3) |
| b/Å | 12.8888(9) | 12.8728(12) | 19.1104(8) | 12.919(3) |
| c/Å | 13.7232(10) | 13.7227(15) | 36.0158(15) | 14.978(3) |
| α/° | 90 | 90 | 90 | 113.20(3) |
| β/° | 90 | 90 | 90 | 103.20(3) |
| γ/° | 90 | 90 | 90 | 102.16(3) |
| Volume/Å^3^ | 2279.7(4) | 2274.0(5) | 8778.6(7) | 2079.0(9) |
| Z | 4 | 4 | 4 | 1 |
| ρ_calc_g/cm^3^ | 1.263 | 1.266 | 1.416 | 1.537 |
| μ/mm^‑1^ | 0.702 | 0.704 | 0.886 | 1.025 |
| F(000) | 920.0 | 920.0 | 3872.0 | 984.0 |
| Crystal size/mm^3^ | 0.5 × 0.07 × 0.07 | 0.5 × 0.06 × 0.06 | 0.1 × 0.05 × 0.05 | 0.32 × 0.22 × 0.17 |
| Radiation | CuKα (λ = 1.54178) | CuKα (λ = 1.54178) | MoKα (λ = 0.71073) | MoKα (λ = 0.71073) |
| 2Θ range for data collection/° | 9.414 to 132.432 | 9.42 to 132.114 | 3.108 to 52 | 3.484 to 49.998 |
| Index ranges | -14 ≤ h ≤ 15, -13 ≤ k ≤ 13, -14 ≤ l ≤ 16 | -14 ≤ h ≤ 12, -15 ≤ k ≤ 14, -12 ≤ l ≤ 15 | -15 ≤ h ≤ 15, -13 ≤ k ≤ 23, -44 ≤ l ≤ 25 | -15 ≤ h ≤ 15, -15 ≤ k ≤ 15, -15 ≤ l ≤ 17 |
| Reflections collected | 10775 | 7377 | 34268 | 21880 |
| Independent reflections | 3706 [R_int_ = 0.0438, R_sigma_ = 0.0498] | 2945 [R_int_ = 0.0341, R_sigma_ = 0.0380] | 17244 [R_int_ = 0.0417, R_sigma_ = 0.0828] | 11091 [R_int_ = 0.0198, R_sigma_ = 0.0250] |
| Data/restraints/parameters | 3706/1/301 | 2945/1/301 | 17244/10/1134 | 11091/3/1111 |
| Goodness-of-fit on F^2^ | 1.063 | 1.065 | 1.011 | 1.053 |
| Final R indexes [I>=2σ (I)] | R_1_ = 0.0289, wR_2_ = 0.0722 | R_1_ = 0.0283, wR_2_ = 0.0741 | R_1_ = 0.0461, wR_2_ = 0.0925 | R_1_ = 0.0203, wR_2_ = 0.0493 |
| Final R indexes [all data] | R_1_ = 0.0296, wR_2_ = 0.0726 | R_1_ = 0.0285, wR_2_ = 0.0744 | R_1_ = 0.0685, wR_2_ = 0.1001 | R_1_ = 0.0203, wR_2_ = 0.0493 |
| Largest diff. peak/hole / e Å^-3^ | 0.11/-0.14 | 0.12/-0.16 | 0.64/-0.70 | 0.33/-0.36 |
| Flack parameter | 0.01(7) | -0.02(7) | 0.018(5) | -0.027(3) |

Table S4. Lifetimes of the **L1 and L2_3_**.Tb complexes in MeOH and MeOD and the corresponding number of solvent molecules coordinated to the lanthanide metal ion. See discussion on the numbers for **L2** in the main body of the article.

| **Complex** | **τ(MeOH) / ms** | **τ(MeOD) / ms** | **q(±0.5)** |
| --- | --- | --- | --- |
| [**(L1a(R,R))_3_**.Tb(OTf)_3_] | 1.703 | 2.086 | 0.65 |
| [**(L1b(S,S))_3_**.Tb(OTf)_3_] | 1.693 | 2.008 | 0.53 |
| [**(L2a(R,R))_3_**.Tb(OTf)_3_] | 1.025 | 1.495 | 2.35 |
| [**(L2b(S,S))_3_**.Tb(OTf)_3_] | 0.976 | 1.596 | 3.09 |

Table S5. g_lum_ values determined for the Tb(III) pair of enantiomers of **L1**

| **Wavelength, nm** | **^5^D_4_→^7^F_J_** | **g_lum_(L1b(SS))** | **g_lum_(L1a(RR))** |
| --- | --- | --- | --- |
| 489.8 | ^5^D_4_→^7^F_6_ | 0.03 | -0.03 |
| 492.4 |  | -0.06 | 0.06 |
| 542.6 | ^5^D_4_→^7^F_5_ | 0.17 | -0.11 |
| 543.8 |  | -0.05 | 0.05 |
| 547.1 |  | 0.44 | -0.50 |
| 549.7 |  | -0.14 | 0.13 |
| 554.6 |  | -0.36 | 0.33 |
| 581.7 | ^5^D_4_→^7^F_4_ | -0.10 | 0.07 |
| 585.7 |  | -0.10 | 0.10 |
| 588.07 |  | 0.04 | -0.04 |
| 596.2 | ^5^D_4_→^7^F_3_ | -0.11 | 0.08 |
| 619.7 |  | -0.35 | 0.31 |
| 623.3 |  | -0.18 | 0.17 |
| 625.3 |  | 0.50 | -0.44 |
| 629.2 |  | -0.18 | 0.12 |

Table S6. Binding constants obtained from fitting the UV-vis and phosphorescence titrations using the non-linear regression analysis program SPECFIT.

|  | **UV-vis** | | | **Phosphorescence** | | |
| --- | --- | --- | --- | --- | --- | --- |
|  | ***β*_1:1_** | ***β*_1:2_** | ***β*_1:3_** | ***β*_1:1_** | ***β*_1:2_** | ***β*_1:3_** |
| **L1a** | **6.45 (±0.16)** | **13.01 (±0.27)** | **18.36 (±0.46)** | **6.96 (±0.13)** | **13.11 (±0.20)** | **19.01 (±0.34)** |
| **L1b** | **6.21**  **(±0.35)** | **12.81**  **(±0.50)** | **18.42**  **(±0.75)** | **6.50**  **(±0.07)** | **12.37**  **(±0.09)** | **18.42**  **(±0.21)** |
| **L2a** | **6.63**  **(±0.26)** | **13.38**  **(±0.41)** | **18.49**  **(±0.66)** | **7.06**  **(±0.11)** | **13.21**  **(±0.17)** | **19.11**  **(±0.29)** |
| **L2b** | **6.75**  **(±0.26)** | **13.24**  **(±0.40)** | **19.03**  **(±0.58)** | **7.12**  **(±0.09)** | **13.02**  **(±0.14)** | **19.30**  **(±0.23)** |
